# Exogenous 24-Epibrassinolide Alleviates Postharvest Chilling Injury in Green Pepper Fruit Associated with Enhanced Antioxidant Defense and Reduced Membrane Damage

**DOI:** 10.3390/plants15192931

**Published:** 2026-09-24

**Authors:** Yanan Ren, Patiguli Wumaier, Yaxin Ge, Jinyang Li, Xuena Rang, Zhifeng Zhu, Wei Dong, Mingjing Zhang, Yuquan Duan

**Affiliations:** 1 Key Laboratory of Agro-Products Quality and Safety Control in Storage and Transport Process, Ministry of Agriculture and Rural Affairs Institute of Food Science and Technology, Chinese Academy of Agricultural Sciences, Beijing 100193, China; 15536508826@163.com (Y.R.); 15699415792@163.com (P.W.); geyaxinn@163.com (Y.G.); 15998244126@163.com (J.L.); rangxuena@163.com (X.R.);; 2 Department of Food Science, Shenyang Agricultural University, Shenyang 110866, China

**Keywords:** 24-epibrassinolide, green pepper, chilling injury, antioxidant defense, reactive oxygen species, membrane damage, transcriptome

## Abstract

Green pepper is highly susceptible to chilling stress during extended low-temperature storage. This study examined the physiological responses and associated transcriptional changes in postharvest green pepper fruit following exogenous 24-epibrassinolide (EBR) treatment. After treatment with 15 μmol·L^−1^ EBR, fruits were maintained at 4.0 ± 1.0 °C for 25 d. Chilling injury, quality attributes, reactive oxygen species (ROS) accumulation, enzymatic and non-enzymatic antioxidant defenses, and membrane damage-related parameters were monitored, and transcriptomic profiling was performed at 25 d. EBR treatment reduced chilling injury and weight loss and delayed softening and storage-related quality deterioration. It also helped maintain antioxidant defenses, limited O_2_•^−^ production and H_2_O_2_ and malondialdehyde (MDA) accumulation, and lowered relative electrical conductivity and phospholipase D (PLD) and lipoxygenase (LOX) activities. Transcriptomic analysis identified EBR-associated changes in processes involving cell wall remodeling, stress responses and signaling, ROS production, and membrane lipid-related functions. These results indicate that improved chilling tolerance in EBR-treated green pepper is closely associated with sustained antioxidant defenses, lower ROS accumulation, and reduced membrane damage, while the transcriptomic data provide additional molecular clues linked to these physiological responses.

## 1. Introduction

Green pepper (*Capsicum annuum* L.) is widely consumed as a fresh vegetable and is valued for its vitamin C, carotenoids, polyphenols, and other bioactive compounds [1,2]. Its high moisture content, however, also makes the fruit highly perishable after harvest. Water loss, discoloration, decay, and nutritional deterioration can develop rapidly during storage and distribution [3]. Refrigeration is commonly used to slow respiration and microbial deterioration, but green pepper remains vulnerable to injury during prolonged low-temperature storage [4,5]. Once chilling injury develops, symptoms such as surface pitting, water-soaked patches, tissue softening, and decay can markedly reduce visual quality and marketability [6,7]. Improving cold-storage tolerance without losing the preservation benefits of refrigeration therefore remains an important issue for postharvest green pepper.

A major feature of chilling stress is disruption of the balance between reactive oxygen species (ROS) generation and scavenging [8]. Under prolonged low-temperature conditions, superoxide anion (O_2_•^−^) and hydrogen peroxide (H_2_O_2_) may accumulate beyond the scavenging capacity of plant tissues [9,10,11]. Such an imbalance can disturb cellular redox status and accelerate oxidative damage to membrane lipids, which is often accompanied by increased malondialdehyde (MDA) levels and greater membrane permeability [10,12]. Green pepper fruit relies on both enzymatic and non-enzymatic defenses to limit this damage. Superoxide dismutase (SOD), catalase (CAT), peroxidase (POD), and ascorbate peroxidase (APX) participate in ROS detoxification, while glutathione (GSH), ascorbic acid, and phenolic compounds provide additional antioxidant buffering [13,14,15,16]. These components were therefore examined together to assess whether EBR-associated chilling tolerance was accompanied by improved redox regulation.

Membrane deterioration is another important component of chilling injury. Low temperature can alter membrane organization and lipid behavior, leading to increased permeability and impaired cellular function [13,17]. In bell pepper, chilling injury has been associated with changes in membrane lipid composition as well as increases in electrolyte leakage, MDA accumulation, and phospholipase D (PLD) activity [17,18]. PLD contributes to phospholipid breakdown, whereas lipoxygenase (LOX) catalyzes the oxidation of polyunsaturated fatty acids. Elevated activities of these enzymes are therefore often linked with enhanced lipid degradation and peroxidative damage [19,20,21]. Conversely, treatments that lessen chilling injury in green pepper and other horticultural products frequently coincide with lower PLD and LOX activities and improved membrane stability [22,23,24]. These observations suggest that limiting membrane lipid deterioration is an important component of cold tolerance.

Brassinosteroids are steroidal plant hormones that participate in growth, development, and stress adaptation [25,26]. Among them, 24-epibrassinolide (EBR) has been widely used as an exogenous regulator in studies of postharvest stress tolerance. Protective effects of EBR under cold-storage conditions have been documented in blood orange, peach, and pomegranate, where treated fruit generally showed less chilling injury, improved antioxidant status, and reduced membrane deterioration [27,28,29]. In blood orange, for instance, EBR treatment was associated with lower electrolyte leakage and better retention of phenolic compounds and antioxidant activity [27]. Similar protective responses have been reported in peach, including changes in membrane lipid-related processes [23]. Brassinosteroid treatment has also recently been reported to improve postharvest quality in bell pepper [30]. These findings suggest that EBR may alleviate chilling injury in postharvest green pepper fruit. However, whether EBR can effectively mitigate chilling injury in green pepper during cold storage, and how the associated physiological and transcriptional responses are coordinated, remains unclear.

Transcriptomic responses to EBR have been investigated in pepper seedlings [31], but comparable information for harvested green pepper fruit remains limited. In particular, the relationship between EBR-associated transcriptional changes and the physiological responses of ROS metabolism, antioxidant defense, and membrane damage in postharvest fruit is still not well defined. We therefore hypothesized that EBR treatment could alleviate chilling injury in postharvest green pepper fruit and that this response would be accompanied by enhanced antioxidant defense, reduced ROS accumulation and membrane damage, and corresponding transcriptional changes.

To test this hypothesis, green pepper fruit was treated with 15 μmol·L^−1^ EBR and monitored throughout 25 d of cold storage. The specific objectives were to (i) evaluate the effects of EBR on chilling injury and quality changes during cold storage; (ii) characterize the associated changes in ROS accumulation, antioxidant responses, and membrane damage-related parameters; and (iii) examine EBR-associated transcriptional changes at 25 d, with particular attention to cell wall remodeling, stress responses/signaling, ROS production, and membrane lipid-related processes. By integrating these physiological and transcriptomic observations, this study aimed to provide a broader view of the responses associated with EBR-enhanced chilling tolerance in postharvest green pepper fruit. A complete list of abbreviations used in this manuscript and the Supplementary Materials is provided in Table S4.

## 2. Results

### 2.1. Chilling Injury Symptoms and Quality Changes During Cold Storage

Figure 1A shows the progressive appearance changes in green pepper fruit in the control (CK) and EBR treatments as storage time increased. Compared with the CK group, EBR-treated fruit generally maintained a better appearance toward the end of storage. In particular, following 25 d of cold storage and a further 3 d of shelf life, wrinkling and overall appearance deterioration were less pronounced in EBR-treated fruit.

The chilling injury (CI) index rose with storage duration in both groups (Figure 1B). Significant differences between CK- and EBR-treated fruit were detected from 10 to 25 d (adjusted *p* < 0.05). At 15 d, CI reached 29.50% in CK fruit but 22.17% in EBR-treated fruit. The difference between treatments increased at 20 d, when the corresponding values were 50.00% and 36.50%, and remained evident at 25 d (64.00% versus 45.00%). These results indicate that EBR treatment slowed the development of chilling injury during cold storage.

Firmness declined in both groups as storage progressed (Figure 1C). The initial value was 24.56 N, whereas by 25 d it had fallen to 12.43 N in CK fruit. EBR-treated fruit retained a significantly higher firmness of 15.93 N at 25 d (adjusted *p* < 0.05), indicating that EBR treatment delayed firmness loss during cold storage. Weight loss increased steadily during storage (Figure 1D). By 25 d, CK fruit had lost 6.68% of their initial mass, compared with 4.71% in the EBR-treated group (adjusted *p* < 0.05). This difference indicates that EBR treatment helped limit water loss during storage.

Color parameters also changed markedly during cold storage (Figure 1E–G). The L* value generally increased with storage duration, and EBR-treated fruit showed significantly lower L* values than CK fruit from 5 to 25 d (adjusted *p* < 0.05), indicating that EBR treatment slowed storage-associated color changes (Figure 1E). The a* value also varied during storage (Figure 1F). Significant differences between CK- and EBR-treated fruit were observed at 5, 10, and 20 d (adjusted *p* < 0.05), whereas no significant differences were detected at 15 and 25 d (adjusted *p* > 0.05). The b* value increased continuously with storage duration and reached 35.61 and 32.30 in the CK- and EBR-treated groups, respectively, at 25 d. The lower b* value in EBR-treated fruit (adjusted *p* < 0.05) indicated a slower increase in yellowness during storage (Figure 1G). Total soluble solids (TSS) content generally decreased during storage (Figure 1H). The TSS content of the CK group decreased from an initial value of 4.39 °Brix to 3.77 °Brix at 25 d, whereas EBR-treated fruit retained a significantly higher value of 3.92 °Brix at the same time point (adjusted *p* < 0.05), indicating that EBR treatment delayed the decline in TSS content during cold storage. Overall, treatment with 15 μmol·L^−1^ EBR reduced chilling injury and weight loss and delayed softening, color changes, and the decline in TSS content during cold storage, thereby helping to maintain the postharvest quality of green pepper fruit.

### 2.2. ROS Accumulation and Oxidative Damage During Cold Storage

Low-temperature storage induced marked changes in reactive oxygen species (ROS) accumulation and oxidative damage in green pepper fruit. The O_2_•^−^ production rate followed different patterns in the two treatments (Figure 2A). CK fruit showed a continuous rise from 3.20 μmol·min^−1^·kg^−1^ at 0 d to 6.15 μmol·min^−1^·kg^−1^ at 25 d. In EBR-treated fruit, the rate first declined at 5 d and then increased gradually. From 5 d onward, however, values in the EBR group remained below those in CK fruit at every sampling point (adjusted *p* < 0.05). The difference between the two treatments became more pronounced as storage progressed.

Changes in H_2_O_2_ content showed a similar overall pattern (Figure 2B). H_2_O_2_ content in CK fruit increased progressively from 0.53 μmol·g^−1^ at 0 d to 2.45 μmol·g^−1^ at 25 d. During the early stage of storage, no significant difference in H_2_O_2_ content was observed between CK- and EBR-treated fruit at 5 d (adjusted *p* > 0.05). From 10 d onward, however, H_2_O_2_ content in EBR-treated fruit was significantly lower than that in CK fruit (adjusted *p* < 0.05). At 25 d, H_2_O_2_ content reached 1.87 μmol·g^−1^ in EBR-treated fruit, representing a reduction of approximately 23.7% compared with the CK group. These results showed that EBR treatment reduced H_2_O_2_ accumulation, particularly during the middle and late stages of cold storage. MDA content was further determined to evaluate lipid peroxidation and oxidative damage (Figure 2C). In CK fruit, MDA content increased continuously during storage, from 1.58 nmol·g^−1^ at 0 d to 3.21 nmol·g^−1^ at 25 d. Although MDA content also increased in EBR-treated fruit, its level remained significantly lower than that in CK fruit from 5 to 25 d (adjusted *p* < 0.05). At the end of storage, MDA content in EBR-treated fruit was 2.85 nmol·g^−1^, approximately 11.2% lower than that in CK fruit. The lower MDA accumulation in EBR-treated fruit was consistent with reduced lipid peroxidation during prolonged cold storage.

Prolonged cold storage was accompanied by increased O_2_•^−^ production, H_2_O_2_ accumulation, and MDA content in green pepper fruit, whereas EBR treatment significantly attenuated these changes. The differences between CK- and EBR-treated fruit were particularly evident during the middle and late stages of storage, indicating lower oxidative stress in EBR-treated fruit during prolonged cold storage. To further examine the antioxidant responses associated with these changes, the enzymatic and non-enzymatic antioxidant defense systems were subsequently analyzed.

### 2.3. Antioxidant Defense Responses During Cold Storage

#### 2.3.1. Antioxidant Enzyme Activities

Excessive ROS accumulation during cold storage can disturb redox balance in fruit tissues. The activities of SOD, CAT, POD, and APX were therefore examined to evaluate antioxidant enzyme responses to EBR treatment.

SOD activity changed throughout the storage period (Figure 3A). In CK fruit, SOD activity increased during the early storage period, reaching 186.10 U·g^−1^ at 10 d, and then gradually declined to 140.71 U·g^−1^ by 25 d. In EBR-treated fruit, SOD activity continued to increase during the middle stage, reaching its highest level at 15 d before gradually declining. Despite this decline, the EBR group retained higher SOD activity than CK at 15, 20, and 25 d (adjusted *p* < 0.05). At 25 d, SOD activity in EBR-treated fruit remained at 191.15 U·g^−1^, indicating better maintenance of SOD activity during late storage.

CAT activity also changed markedly during storage (Figure 3B). In CK fruit, CAT activity rose during the early stage and then declined substantially, decreasing from 218.42 U·g^−1^ at 10 d to 87.55 U·g^−1^ at 25 d. In EBR-treated fruit, CAT activity declined more slowly after reaching a relatively high level during the early stage, and CAT activity in the EBR group exceeded the CK level from 10 to 25 d (adjusted *p* < 0.05). At 25 d, CAT activity in EBR-treated fruit was 168.33 U·g^−1^, indicating a slower decline in CAT activity after EBR treatment. A general decline in POD activity occurred in both treatments (Figure 3C). CK fruit decreased from 289.07 U·g^−1^ at 0 d to 53.57 U·g^−1^ at 25 d. EBR-treated fruit followed a comparable downward pattern, although the reduction was somewhat slower. Significant treatment differences were detected only at 10 and 20 d, when POD activity was higher in EBR-treated fruit (adjusted *p* < 0.05). At the other sampling times, no treatment effect was detected, indicating that the POD response to EBR was stage-dependent. APX activity also tended to decline as storage proceeded (Figure 3D). Compared with CK, EBR-treated fruit retained higher APX activity at 5, 10, 15, and 25 d (adjusted *p* < 0.05), whereas the two groups did not differ significantly at 20 d. Although APX activity decreased substantially in both treatments by 25 d, it remained higher in EBR-treated fruit, indicating better maintenance of APX activity during late storage.

#### 2.3.2. Non-Enzymatic Antioxidants

GSH, phenolic compounds, and vitamin C (VC) also contribute to non-enzymatic antioxidant defense and cellular redox balance. Their responses during storage were therefore examined. GSH content varied with storage stage (Figure 3E). CK fruit showed an early increase and reached the highest level at 10 d, followed by a sharp reduction at 15 d, a slight recovery at 20 d, and a small decrease at 25 d. In EBR-treated fruit, levels showed smaller fluctuations and were higher than those in CK fruit at 15 and 25 d (adjusted *p* < 0.05). By 25 d, GSH content was 45.23 μmol·g^−1^ in EBR-treated fruit and 38.92 μmol·g^−1^ in CK fruit, showing better retention of GSH in the EBR group during late storage.

Total phenolic content showed pronounced fluctuations during storage (Figure 3F). At 5 d, total phenolic content increased sharply in CK fruit and was significantly higher than that in EBR-treated fruit (adjusted *p* < 0.05), after which it declined rapidly. The two treatments did not differ at 10 d. At 15 d, total phenolic content was significantly higher in EBR-treated fruit than in CK fruit (adjusted *p* < 0.05). No treatment difference was detected at 20 d, whereas EBR-treated fruit again showed a significantly higher total phenolic content at 25 d (adjusted *p* < 0.05). Overall, phenolic levels were better maintained in EBR-treated fruit during the middle and late storage stages. VC declined progressively during storage (Figure 3G). VC levels were comparable between treatments at 5 d. From 10 d onward, EBR-treated fruit consistently retained more VC than CK (adjusted *p* < 0.05). At 25 d, VC content had decreased to approximately 0.39 g·kg^−1^ in CK fruit, whereas EBR-treated fruit retained approximately 0.60 g·kg^−1^. This pattern indicates slower VC loss following EBR treatment.

The enzymatic and non-enzymatic antioxidant components of green pepper fruit showed distinct dynamic changes during cold storage. Compared with CK fruit, EBR treatment generally maintained higher levels of SOD, CAT, APX, GSH, total phenolics, and VC during the middle and late stages of storage. These antioxidant responses were consistent with lower O_2_•^−^ production and reduced H_2_O_2_ and MDA accumulation observed in EBR-treated fruit, indicating better maintenance of antioxidant defenses during prolonged cold storage.

### 2.4. Membrane Integrity and Lipid-Related Responses During Cold Storage

Cold storage caused marked changes in membrane-related parameters of green pepper fruit. As shown in Figure 4A, relative electrical conductivity in CK fruit increased continuously with storage duration, from 5.44% at 0 d to 23.15% at 25 d. The increase was smaller after EBR treatment, and conductivity remained below the CK level from 5 to 25 d (adjusted *p* < 0.05). At 25 d, relative electrical conductivity in EBR-treated fruit was 18.83%, approximately 18.7% lower than that in CK fruit, indicating better maintenance of membrane integrity after EBR treatment.

PLD activity varied nonlinearly during storage (Figure 4B). In CK fruit, PLD activity increased rapidly from 38.20 U·g^−1^ at 0 d to 65.44 U·g^−1^ at 10 d, followed by a slight decline and a subsequent increase to 66.29 U·g^−1^ at 25 d. From 5 to 25 d, PLD activity was consistently lower in EBR-treated fruit than in CK (adjusted *p* < 0.05). The difference between the two groups was more pronounced during the early stage of storage and gradually narrowed as storage progressed; nevertheless, PLD activity in EBR-treated fruit remained significantly lower than that in CK fruit at 25 d. LOX activity also varied during the storage period (Figure 4C). In CK fruit, LOX activity increased from 9.46 U·g^−1^ at 0 d to 15.05 U·g^−1^ at 15 d, followed by a slight decrease, and remained at a relatively high level of 15.02 U·g^−1^ at 25 d. EBR-treated fruit maintained lower LOX activity than CK from 5 to 25 d (adjusted *p* < 0.05). Overall, the increases in relative electrical conductivity, PLD activity, and LOX activity were less pronounced in EBR-treated fruit. Together with the lower MDA accumulation, these responses indicated reduced membrane damage and improved membrane integrity.

### 2.5. Transcriptomic Responses of Green Pepper Fruit to EBR Treatment During Cold Storage

#### 2.5.1. Sample Relationships and Differentially Expressed Gene Analysis

Fruit from the CK and 15 μmol·L^−1^ EBR groups stored for 25 d were selected for comparative transcriptomic analysis based on the development of chilling injury. At this stage, chilling injury was relatively severe and clear phenotypic differences were observed between the two treatments, allowing transcriptional differences associated with EBR treatment to be examined. Each group contained three biological replicates. Across the six RNA sequencing (RNA-seq) libraries, 21.87–25.51 million raw reads and 21.18–24.29 million clean reads were obtained per sample, corresponding to 6.22–7.04 Gb of clean bases. The percentage of bases with a Phred quality score ≥ 30 (Q30) ranged from 94.52% to 95.03%, overall mapping rates from 97.19% to 97.23%, uniquely mapped reads from 94.39% to 94.56%, and RNA integrity number (RIN) values from 9.1 to 9.5. Detailed RNA-seq quality-control metrics for all samples are provided in Table S2.

In the principal component analysis (PCA), PC1 accounted for 53.68% of the variance and PC2 for 25.10%, giving a cumulative contribution of 78.78% (Figure 5A). CK- and EBR-treated samples were separated mainly along PC1, reflecting differences in their overall transcriptional profiles after 25 d of cold storage. Although some within-group variation was observed among biological replicates, CK- and EBR-treated samples remained clearly separated mainly along PC1. Pearson correlation coefficients were generally high among replicates (Figure 5B), ranging from 0.97 to 0.98 in the EBR-treated group and from 0.93 to 0.99 in the CK group, indicating overall good consistency among the RNA-seq samples.

Applying an adjusted *p*-value (padj) < 0.05 and |log_2_(fold change)| > 1 identified 723 differentially expressed genes (DEGs) between CK- and EBR-treated fruit, including 273 upregulated and 450 downregulated genes (Figure 5C). The DEG analysis revealed clear transcriptional differences between CK- and EBR-treated fruit after prolonged cold storage.

#### 2.5.2. GO Enrichment Analysis and Candidate Gene Expression Patterns

Gene Ontology (GO) enrichment was examined separately for the upregulated and downregulated DEG sets. Twelve GO terms were significantly enriched among the upregulated DEGs (Benjamini–Hochberg [BH]-adjusted *p*-value < 0.05), mainly involving xyloglucan metabolic process, cell wall organization, cell wall biogenesis, and xyloglucan:xyloglucosyl transferase activity (Figure 5D). These enriched terms were mainly related to cell wall remodeling.

The downregulated DEG set contained 47 significantly enriched GO terms, mainly involving response to cold, response to osmotic stress, calcium-mediated signaling, calcium ion transport, phospholipase activity, galactolipase activity, and unsaturated fatty acid biosynthetic process (Figure 5E). These terms point to differences between CK- and EBR-treated fruit in stress-related signaling and membrane lipid-related processes.

Candidate genes were further examined in relation to the physiological responses observed during cold storage (Figure 5F). These genes were mainly associated with cell wall remodeling, ROS production, membrane lipid-related processes, and stress signaling. Several xyloglucan endotransglucosylase/hydrolase (*XTH*)-related genes showed higher transcript abundance in EBR-treated fruit, whereas ROS production-related genes, including *RBOHC* and *RBOHE*, and membrane lipid-related genes, including *PLDBETA1*, *LOX3.1*, and several fatty acid desaturase (*FAD*)-related genes, showed lower transcript abundance than in CK fruit. Detailed differential-expression statistics for the candidate genes shown in Figure 5F are provided in Table S3. Overall, these expression patterns were broadly consistent with the physiological differences observed in ROS accumulation, antioxidant defense, membrane integrity, and chilling injury.

The transcriptomic results further indicate EBR-associated transcriptional changes involving cell wall remodeling, stress responses, ROS production, and membrane lipid-related processes.

## 3. Discussion

Green pepper is a typical chilling-sensitive horticultural commodity. Prolonged exposure to unsuitable low temperatures can lead to surface pitting, water loss, softening, and decay, together with declines in nutritional quality and commercial value. Previous studies have shown that brassinosteroid treatments can improve chilling tolerance in green pepper and other postharvest fruits [27,28,29,30]. In the present study, treatment with 15 μmol·L^−1^ EBR significantly reduced the chilling injury index of green pepper during the middle and late stages of cold storage, reduced weight loss, delayed firmness loss, and better maintained color, total soluble solids, and vitamin C content. These findings suggest that EBR not only delayed the development of visible chilling symptoms, but also limited water loss, tissue softening, and overall quality decline. Similar protective effects have been reported in other chilling-sensitive fruits, where EBR reduced chilling injury and electrolyte leakage in blood orange and helped preserve postharvest quality and antioxidant capacity in peach and pomegranate. These studies, together with our results, support a protective effect of EBR in chilling-sensitive fruits.

Disturbance of reactive oxygen species (ROS) metabolism is an important physiological feature of chilling injury in fruits and vegetables. In CK fruit, the O_2_•^−^ production rate and H_2_O_2_ content increased continuously with storage time and were accompanied by progressive MDA accumulation, indicating that prolonged low-temperature storage disrupted redox homeostasis and intensified lipid peroxidation. EBR treatment markedly suppressed O_2_•^−^ and H_2_O_2_ accumulation during the middle and late stages of storage and also reduced MDA levels, indicating a lower oxidative burden under chilling conditions. Similar responses have been reported in blood orange, peach, and pomegranate fruit [27,28,29]. ROS homeostasis in plant tissues is jointly regulated by enzymatic and non-enzymatic antioxidant systems. SOD converts O_2_•^−^ into H_2_O_2_, while CAT, POD, and APX contribute to the subsequent removal of H_2_O_2_. GSH, phenolic compounds, and vitamin C also participate directly or indirectly in redox cycling and help maintain the intracellular reducing environment [13,15]. In the present study, several antioxidant enzymes in CK fruit declined to varying degrees during the middle and late stages of storage, whereas EBR-treated fruit maintained higher SOD, CAT, and APX activities at several sampling points. The response of POD was less consistent, with significant differences observed only at 10 and 20 d. EBR-treated fruit also generally retained higher levels of GSH, total phenolics, and vitamin C during the later storage period. Notably, total phenolic content was transiently higher in CK fruit at 5 d. This early increase may represent a rapid stress-associated phenolic response in untreated fruit, whereas the subsequent pattern differed between treatments. These antioxidant responses were consistent with lower O_2_•^−^ production and reduced H_2_O_2_ and MDA accumulation after EBR treatment, suggesting that maintenance of both enzymatic and non-enzymatic antioxidant defenses may help restrict excessive ROS accumulation during prolonged cold storage.

The cell membrane is one of the structures most susceptible to chilling-induced damage in fruits and vegetables. Low temperature can alter membrane lipid composition, fluidity, and metabolism, eventually compromising membrane structure. Relative electrical conductivity and MDA content are commonly used as indicators of membrane permeability and lipid peroxidation, while PLD and LOX are important enzymes involved in membrane lipid degradation and oxidation. PLD hydrolyzes membrane phospholipids to generate phosphatidic acid, whereas LOX catalyzes the oxidation of polyunsaturated fatty acids and thereby promotes lipid peroxidation and membrane damage. Previous studies have shown that chilling injury in green pepper is accompanied by marked changes in membrane lipid composition, activation of PLD-related processes, and loss of membrane integrity, highlighting the importance of membrane lipid homeostasis for chilling tolerance [16,17]. In this study, relative electrical conductivity and PLD and LOX activities in CK fruit showed an overall increasing trend during storage, whereas these changes were significantly moderated by EBR treatment. The lower PLD and LOX activities in EBR-treated fruit were accompanied by lower MDA content and relative electrical conductivity, consistent with reduced lipid peroxidation and improved membrane preservation. Together with the ROS and antioxidant results, these findings suggest that maintenance of antioxidant status may reduce oxidative pressure, while lower membrane lipid degradation and peroxidation may help preserve membrane stability. These two processes may jointly contribute to the alleviation of chilling injury by EBR.

Transcriptomic analysis provided further transcriptional clues related to these physiological responses. After EBR treatment, upregulated genes were significantly enriched in GO terms associated with xyloglucan metabolism, cell wall organization, and cell wall biogenesis, and several genes annotated as *XTH15* and *XTH16* were significantly upregulated. XTH proteins participate in the cleavage and reconnection of xyloglucan chains and play important roles in cell wall loosening, restructuring, and maintenance [32]. The increased expression of *XTH*-related genes indicates that EBR treatment was accompanied by altered cell wall remodeling. Given the diverse functions of XTH family members in xyloglucan cleavage and transglycosylation, their upregulation should not be interpreted as direct evidence of either cell wall loosening or firmness maintenance. The relationship between these transcriptional changes and the delayed firmness loss observed in EBR-treated fruit requires further validation. Meanwhile, the enrichment of downregulated DEGs in terms related to cold response and calcium-mediated signaling may reflect differences in stress status between CK- and EBR-treated fruit at 25 d rather than direct suppression of these processes by EBR.

Candidate genes related to ROS production and membrane lipid processes also showed expression patterns that paralleled several physiological responses. The ROS production-related genes *RBOHC* and *RBOHE* were significantly downregulated after EBR treatment. Respiratory burst oxidase homolog (RBOH) proteins are plasma membrane nicotinamide adenine dinucleotide phosphate (*NADPH*) oxidases involved in ROS production and stress signaling. Their lower transcript abundance corresponded with the reduced O_2_•^−^ production rate and H_2_O_2_ content observed in EBR-treated fruit, suggesting that transcriptional changes related to ROS generation may be involved in the redox response to EBR treatment. At the same time, *PLDBETA1* and *LOX3.1* were significantly downregulated, in agreement with the lower PLD and LOX activities, MDA accumulation, and relative electrical conductivity. These transcriptional changes were observed alongside the lower degree of membrane damage in EBR-treated fruit. *FAD2* and fatty acid desaturase (*FAD*)-related genes were also downregulated, while GO terms associated with unsaturated fatty acid biosynthesis were enriched among the downregulated genes. These transcriptional changes suggest that EBR treatment was associated with changes in membrane lipid-related processes during cold storage. Given the important role of fatty-acid unsaturation in maintaining membrane fluidity under low-temperature conditions, however, the downregulation of *FAD*-related genes should not be directly interpreted as evidence of improved membrane stability. Instead, when considered together with the lower MDA content, relative electrical conductivity, PLD activity, and LOX activity observed in EBR-treated fruit, these results indicate that EBR treatment was accompanied by parallel physiological and transcriptional changes related to membrane integrity and lipid metabolism. Multi-time-point physiological measurements showed that 24-epibrassinolide (EBR) alleviated chilling injury, reduced reactive oxygen species (ROS) accumulation, maintained antioxidant defense, and limited membrane damage during cold storage. Transcriptomic analysis was conducted at 25 d of storage, when chilling injury and quality deterioration were pronounced, and phenotypic differences between the control (CK) and EBR-treated groups were clearly evident, allowing the relationship between late-stage physiological responses and transcriptional changes to be examined. However, because RNA-seq was performed only at this time point, the observed transcriptional differences may also partly reflect secondary responses associated with different degrees of chilling injury. Therefore, these changes should be interpreted as associative rather than as evidence of a direct causal mechanism.

The physiological and transcriptomic results indicate that treatment with 15 μmol·L^−1^ EBR helped maintain enzymatic and non-enzymatic antioxidant defenses during cold storage, limited ROS accumulation, and reduced lipid peroxidation and membrane damage in green pepper fruit. EBR treatment was also accompanied by changes in the expression of genes related to cell wall remodeling, stress responses, ROS production, and membrane lipid-related processes, several of which showed trends consistent with the physiological observations. Compared with studies focusing mainly on postharvest quality or selected physiological traits, the present work combined multi-time-point measurements of chilling injury and quality, ROS homeostasis, antioxidant defense, and membrane damage with transcriptomic profiling at a representative late stage of cold storage when treatment-related phenotypic differences were clearly evident. This integrated approach provides complementary physiological and transcriptional evidence for the association between antioxidant maintenance, ROS homeostasis, and reduced membrane damage. The findings therefore extend the physiological understanding of EBR-mediated chilling tolerance in postharvest green pepper and provide additional evidence for the close relationship between redox status and membrane stability during low-temperature stress in chilling-sensitive produce. One limitation of the present study is that transcriptomic analysis was conducted at 25 d, and the selected candidate genes were not further validated by reverse transcription quantitative PCR (RT-qPCR). Therefore, the transcriptomic results should be interpreted as associative evidence that complements the physiological findings.

From a postharvest preservation perspective, reducing losses of perishable fruits and vegetables and improving the efficiency of cold-chain utilization are increasingly important for the sustainability of food systems under climate change and growing uncertainty in food supply chains [33,34]. For chilling-sensitive commodities such as green pepper, low-temperature storage can slow quality deterioration, but prolonged exposure to cold also increases the risk of chilling injury and limits the effectiveness of refrigeration. The present results suggest that EBR pretreatment may serve as an auxiliary approach for low-temperature storage of green pepper. Future work should combine temporal gene-expression analysis with fatty acid profiling, lipidomics, and functional characterization of key candidate genes to clarify the processes through which EBR influences ROS homeostasis and membrane stability. Further studies across different cultivars and EBR concentrations, together with evaluation under practical cold-chain transportation and shelf-life conditions, will be necessary to determine the robustness, stability, and economic feasibility of this treatment. Such work would help clarify the practical value of EBR for postharvest management of green pepper and other chilling-sensitive commodities.

## 4. Materials and Methods

### 4.1. Plant Materials and Treatment

Green pepper fruits (*Capsicum annuum* L. cv. Changjian No. 1) at commercial maturity were obtained from a pepper-growing farm in Shouguang, Shandong Province, China, and transported to the laboratory within 24 h after harvest. Fruits with uniform size, bright green color, and similar maturity were selected, while those showing mechanical injury, disease symptoms, or insect damage were discarded.

Based on an initial concentration-screening experiment using 5, 15, and 25 μmol·L^−1^ EBR, together with previous studies [35,36], 15 μmol·L^−1^ EBR was selected for the storage trial because it showed the most consistently favorable overall performance across chilling injury, postharvest quality, antioxidant responses, ROS accumulation, and membrane damage-related parameters within the tested concentration range. EBR (purity: 92%) was purchased from Shanghai Yuanye Bio-Technology Co., Ltd. (Shanghai, China). A 15 mmol·L^−1^ EBR stock solution was prepared in absolute ethanol and diluted with distilled water to 15 μmol·L^−1^ before use. Tween 80 was added to a final concentration of 0.1% (*v*/*v*). The control (CK) solution contained the same final concentrations of absolute ethanol and Tween 80 as the EBR treatment solution but no EBR. Fruits were fully immersed in the corresponding solutions for 10 min at room temperature, air-dried, placed in plastic crates, and stored at 4.0 ± 1.0 °C with 85 ± 5% relative humidity for 25 d.

Each treatment included three independent biological replicates, with approximately 150 fruits in each replicate and about 450 fruits per treatment. Each biological replicate was treated independently from the beginning of the experiment. Fruits within each replicate were separately immersed in the corresponding treatment solution, air-dried, and placed in an individual plastic crate. All crates were stored in the same cold-storage chamber. Crate positions were randomly assigned at the beginning of storage and then remained fixed throughout the storage period. Each crate was regarded as an independent experimental unit. Samples at 0 d were taken from the same fruit batch before treatment and used as the common initial baseline for CK and EBR groups. Subsequent samples were collected after 5, 10, 15, 20, and 25 d of storage. At each sampling time, 10 fruits were randomly selected from each biological replicate. For fruit-level traits, the 10 fruits were measured individually, and their mean was used as the replicate value. For biochemical measurements, equal amounts of tissue from the 10 fruits were pooled to form one sample representing that biological replicate. The pooled tissues were rapidly frozen in liquid nitrogen and stored at −80 °C until analysis. In addition, 15 fruits per treatment were randomly selected after 15, 20, and 25 d of cold storage and held at room temperature for 3 d for qualitative evaluation of external appearance and visible chilling symptoms after rewarming.

### 4.2. Determination of Storage Quality and Chilling Injury

Except for weight loss, fruits used for quality and biochemical measurements were randomly sampled at each designated storage time and were not reused at subsequent time points. At each sampling time, 10 fruits were randomly selected from each biological replicate for chilling injury and fruit-quality measurements. A separate set of labeled fruits was reserved for weight-loss determination and repeatedly weighed throughout storage.

Chilling injury was evaluated based on visible symptoms, including surface pitting, water-soaked lesions, discoloration, shriveling, and decay. Individual fruits were assigned to one of five injury grades according to the proportion of the affected surface: grade 0, no visible injury; grade 1, affected area ≤ 25%; grade 2, affected area > 25% and ≤50%; grade 3, affected area > 50% and ≤75%; and grade 4, affected area > 75% or obvious decay [37]. The chilling injury index was then computed as:

Chilling injury index (%) = [Σ(*i* × *n_i_*)/(4*N*)] × 100

where *i* denotes the injury grade, *n_i_* represents the number of fruits assigned to grade *i*, *N* is the total number examined, and 4 represents the highest injury grade. For each biological replicate, the same 10 fruits were independently scored by five assessors blinded to treatment identity. The five assessor-derived chilling injury indices were averaged to obtain one final value for that biological replicate. Thus, three biological-replicate values were obtained for each treatment at each storage time (*n* = 3).

Weight loss was assessed by repeatedly weighing the same labeled fruits throughout storage [38]. Fruit mass before storage was recorded as *m*_0_, and the mass measured at each sampling time was recorded as *m_t_*. Weight loss was calculated as follows:


Weight loss (%) = [(*m*_0_ − *m_t_*)/*m*_0_] × 100

For statistical analysis, the mean weight-loss value of the labeled fruits within each biological replicate was calculated at each storage time, and the biological replicate rather than the individual fruit was regarded as the experimental unit.

Firmness was determined using a GY-4 handheld fruit firmness tester (Zhejiang Top Cloud-Agri Technology Co., Ltd., Hangzhou, China) fitted with a 3.5-mm-diameter probe. Readings were collected at three positions around the equator of each fruit, and their mean was reported in N [39]. Fruit color was recorded at three equatorial positions with a DS-400 colorimeter (Hangzhou CHNSpec Technology Co., Ltd., Hangzhou, China), and the average L*, a*, and b* values were used for analysis [40].

A PAL-BX/ACID F5 refractometer (ATAGO China Co., Ltd., Guangzhou, China) was used for TSS measurement. Juice obtained from green pepper flesh was mixed thoroughly before measurement, and TSS was expressed as °Brix [41]. Colorimetric determination of vitamin C (VC) was carried out with a commercial kit (A009-1-1, Nanjing Jiancheng Bioengineering Institute, Nanjing, China). Pepper tissue was mixed with 0.9% physiological saline at 1:9 (g:mL) and homogenized on ice to prepare a 10% homogenate. Following centrifugation at 2500 r·min^−1^ for 10 min, the supernatant was retained for measurement. After the color reaction, absorbance was recorded at 536 nm. VC content was obtained from the corresponding standard and reported as g·kg^−1^.

### 4.3. Determination of Oxidative Stress and Antioxidant Defense Parameters

At each sampling point, green pepper tissue was first frozen in liquid nitrogen and then ground to a fine powder. For SOD, POD, and CAT assays, 0.1 g of the powdered tissue was homogenized on ice with 1.0 mL of the extraction reagent supplied with the corresponding commercial kit and centrifuged at 8000× *g* for 10 min at 4 °C. For APX, 0.1 g of tissue was extracted with 1.0 mL of the kit reagent and centrifuged at 12,000× *g* for 20 min at 4 °C. The resulting supernatants were used for the respective assays. Three biological replicates were included for each treatment.

Spectrophotometric methods were applied to assay superoxide dismutase (SOD), peroxidase (POD), catalase (CAT), and ascorbate peroxidase (APX) activities [42]. SOD activity was determined using an AKAO001M-100S kit (Boxbio, Beijing, China) by monitoring nitroblue tetrazolium (NBT) reduction at 560 nm, with one unit defined as the enzyme amount producing 50% inhibition of NBT reduction. POD activity was measured using a BC0095 kit (Solarbio, Beijing, China) in a guaiacol reaction system at 470 nm, and an increase in absorbance at 470 nm of 0.01 per minute was defined as one unit of activity. CAT activity was determined using a BC0205 kit (Solarbio) by monitoring the decrease in absorbance at 240 nm during H_2_O_2_ decomposition. APX activity was measured using an AKVI006M kit (Boxbio) by following ascorbate oxidation at 290 nm. For CAT, one unit of activity was defined as the amount of enzyme required to decompose 1 μmol of H_2_O_2_ per minute. For APX, one unit of activity was defined as the amount of enzyme required to oxidize 1 μmol of ascorbate per minute.

O_2_•^−^ production was determined using a BC1295 kit (Solarbio) by the hydroxylamine method at 530 nm after extraction of 0.1 g tissue with 1.0 mL of extraction reagent and centrifugation at 12,000 rpm for 10 min at 4 °C. H_2_O_2_ content was measured using an AKAO009M kit (Boxbio) by a titanium-based colorimetric method at 415 nm after extraction of 0.1 g tissue with 1.0 mL of pre-cooled acetone and centrifugation at 8000× *g* for 10 min at 4 °C. GSH content was determined using a BC1175 kit (Solarbio) by the 5,5′-dithiobis(2-nitrobenzoic acid) (DTNB) method at 412 nm after extraction of 0.1 g tissue with 1.0 mL of extraction reagent and centrifugation at 12,000× *g* for 10 min at 4 °C. For total phenolics (AKPL016M-100S, Boxbio), 0.1 g of fresh tissue was ground and extracted with 2.0 mL of extraction reagent by sonication at 60 °C and 300 W for 30 min, followed by centrifugation at 12,000× *g* for 10 min, and absorbance was measured at 760 nm using gallic acid for calibration. SOD, POD, CAT, and APX activities are reported in U·g^−1^. All enzyme activities reported as U·g^−1^ in this study are expressed on a fresh-weight basis. O_2_•^−^ production is given as μmol·min^−1^·kg^−1^, while H_2_O_2_ and GSH are reported as μmol·g^−1^. Total phenolics are expressed as mg gallic acid equivalents (GAE)·g^−1^. Unless otherwise specified, all mass-based biochemical parameters in this section are expressed on a fresh-weight basis. Detailed assay conditions and calculation procedures for oxidative stress and antioxidant defense parameters are provided in Table S1.

### 4.4. Determination of Membrane Integrity and Membrane Lipid Damage-Related Parameters

Membrane integrity during cold storage was assessed by relative electrical conductivity, malondialdehyde (MDA) content, phospholipase D (PLD) activity, and lipoxygenase (LOX) activity [43].

Relative electrical conductivity was determined using a conductivity method [44]. Flesh discs were excised from the equatorial region of the fruit using a 9-mm-diameter cork borer and adjusted to a uniform thickness of 4 mm. The discs were rinsed three times with deionized water to remove electrolytes released during cutting. Fifteen discs were immersed in 20 mL of deionized water at 25 °C for 3 h, after which conductivity was recorded as C_1_. The samples were then heated in a boiling water bath for 30 min, cooled to room temperature, and the final conductivity was recorded as C_2_. Relative electrical conductivity was calculated as:

Relative electrical conductivity (%) = (C_1_/C_2_) × 100

MDA was assayed using the thiobarbituric acid (TBA) color reaction with a commercial kit (BC0025, Solarbio, Beijing, China). Approximately 0.1 g of liquid-nitrogen-ground tissue was extracted with 1.0 mL of extraction reagent and centrifuged at 8000× *g* for 10 min at 4 °C. The supernatant was mixed with the TBA-containing working solution and heated at 100 °C for 60 min. Absorbance was measured at 532 and 600 nm using a Spark^®^ multimode microplate reader (Tecan Trading AG, Männedorf, Switzerland). Nonspecific absorbance was corrected as ΔA = ΔA_532_ − ΔA_600_. Based on an MDA molar extinction coefficient of 1.55 × 10^5^ L·mol^−1^·cm^−1^, MDA content was calculated as 53.763 × ΔA/W × F, where W is the sample mass (g), and F is the dilution factor. MDA values are reported as nmol·g^−1^, with g referring to fresh tissue weight.

PLD and LOX were determined using commercial plant sandwich enzyme-linked immunosorbent assay (ELISA) kits according to the kit protocols. For each assay, each sample well received 40 μL of sample diluent and 10 μL of sample, yielding a fivefold final dilution. The plates were incubated at 37 °C for 30 min and washed five times, followed by the addition of 50 μL of enzyme conjugate and a second incubation at 37 °C for 30 min. After another five washing cycles, 50 μL each of chromogenic solutions A and B were added, and the plates were incubated in the dark at 37 °C for 10 min. After addition of 50 μL of stop solution, absorbance was read at 450 nm within 15 min. PLD and LOX values were calculated from the corresponding standard curves, corrected for the dilution factor, normalized to tissue fresh weight, and expressed as U·g^−1^, with g referring to fresh tissue weight.

### 4.5. Transcriptome Sequencing and Bioinformatics Analysis

Comparative RNA sequencing was conducted on CK and 15 μmol·L^−1^ EBR-treated green pepper fruit collected after 25 d of cold storage. Each treatment contained three biological replicates, resulting in six samples in total. The tissue was immediately frozen in liquid nitrogen and maintained at −80 °C before being sent to Shanghai OE Biotech Co., Ltd. (Shanghai, China), where RNA extraction, library preparation, sequencing, and bioinformatics processing were completed.

Total RNA was extracted using a plant RNA extraction kit based on a column purification method (Cat. No. T105096). RNA concentration and purity were assessed using a NanoDrop 2000 spectrophotometer (Thermo Scientific, Waltham, MA, USA), and RNA integrity was evaluated using an Agilent 2100 Bioanalyzer (Agilent Technologies, Santa Clara, CA, USA). Samples that passed the quality assessment were used for subsequent library preparation and sequencing.

Raw FASTQ reads were processed with fastp v0.20.1 [45] to obtain clean reads. The retained reads were mapped to the *Capsicum annuum* reference genome NCBI_UCD10Xv1.1 [46] with HISAT2 v2.1.0 [47], and gene-level read counts were generated using HTSeq-count v0.11.2 [48].

Sample-level relationships and replicate consistency were examined by PCA and Pearson correlation analysis. DESeq2 v1.22.2 [49] was used for differential expression testing. *p*-values were adjusted for multiple testing using the Benjamini–Hochberg (BH) procedure, and the resulting adjusted *p*-values correspond to the q-values reported in the original analysis. Genes with an adjusted *p*-value (padj) < 0.05 and |log_2_(fold change)| > 1 were considered differentially expressed genes (DEGs).

Gene Ontology (GO) enrichment was analyzed separately for upregulated and downregulated DEGs [50], with GO-annotated genes in the reference genome used as the background set. *p*-values from the GO enrichment analysis were adjusted for multiple testing using the Benjamini–Hochberg (BH) procedure, and terms with an adjusted *p*-value < 0.05 were regarded as significantly enriched. Candidate genes for the heatmap were selected from the significant DEG set (padj < 0.05 and |log_2_(fold change)| > 1) based on their functional annotations and their relevance to the physiological processes examined in this study, including cell wall remodeling, stress responses and signaling, ROS production, and membrane lipid-related processes. Their expression patterns in CK- and EBR-treated samples were displayed as a heatmap.

### 4.6. Statistical Analysis

Each experiment comprised three biological replicates, with each plastic crate regarded as an independent experimental unit, and results are reported as the mean ± standard error (SE). IBM SPSS Statistics 26.0 was used for statistical testing, whereas Origin 2024 was used to prepare the figures. For variables measured during storage, a two-way mixed-design repeated-measures analysis of variance (ANOVA) was used to evaluate the effects of treatment, storage time, and their interaction (treatment × storage time). Samples collected at 0 d were obtained from the same batch of fruit before treatment and were treated as a common baseline; therefore, they were excluded from treatment-related inferential analyses. To characterize treatment differences at individual storage times, comparisons between CK and EBR were performed with Bonferroni adjustment for multiple comparisons. Differences were considered statistically significant at adjusted *p* < 0.05. At the same storage time, different lowercase letters indicate significant differences between the control (CK) and EBR treatments, whereas identical letters indicate no significant difference. Principal component analysis (PCA), Pearson correlation analysis, and transcriptomic correlation heatmaps were produced on the OECloud platform (Shanghai OE Biotech Co., Ltd., Shanghai, China; https://cloud.oebiotech.com/; accessed on 22 March 2026).

## 5. Conclusions

Treatment with 15 μmol·L^−1^ EBR alleviated chilling injury in green pepper fruit during cold storage, reduced weight loss, and delayed softening, color deterioration, and postharvest quality loss. EBR-treated fruit better maintained enzymatic and non-enzymatic antioxidant defenses and showed lower O_2_•^−^ production, H_2_O_2_ and MDA accumulation, relative electrical conductivity, and PLD and LOX activities. These physiological responses indicate that maintenance of antioxidant defense and reductions in oxidative stress and membrane damage are closely associated with the improved chilling tolerance of EBR-treated fruit. Transcriptomic analysis further identified EBR-associated transcriptional changes involving cell wall remodeling, stress signaling, ROS production, and membrane lipid-related processes, with several candidate genes showing expression patterns consistent with these physiological responses. Overall, these findings provide physiological and transcriptional evidence for EBR-associated chilling tolerance in postharvest green pepper fruit and suggest the potential of 15 μmol·L^−1^ EBR as an auxiliary treatment for chilling injury mitigation and postharvest quality maintenance under the conditions tested in this study.

## Figures and Tables

**Figure 1:**
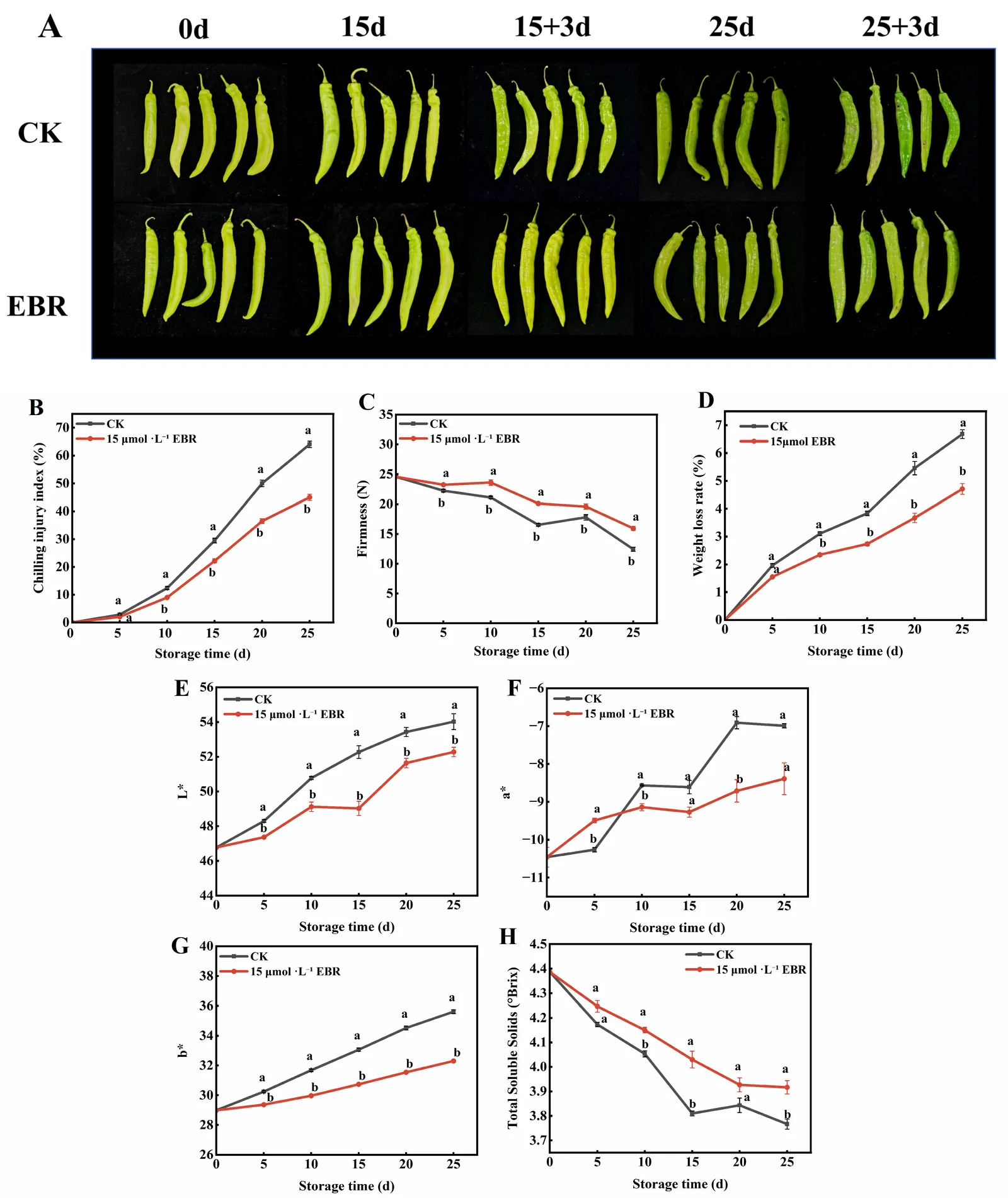
Chilling injury symptoms and quality changes during cold storage. (**A**) Fruit appearance. (**B**) Chilling injury index. (**C**) Firmness. (**D**) Weight loss. (**E**) L* value. (**F**) a* value. (**G**) b* value. (**H**) Total soluble solids content. Values are expressed as mean ± standard error (SE) (*n* = 3). Different lowercase letters at the same storage time indicate significant differences between treatments after Bonferroni adjustment (adjusted *p* < 0.05).

**Figure 2:**
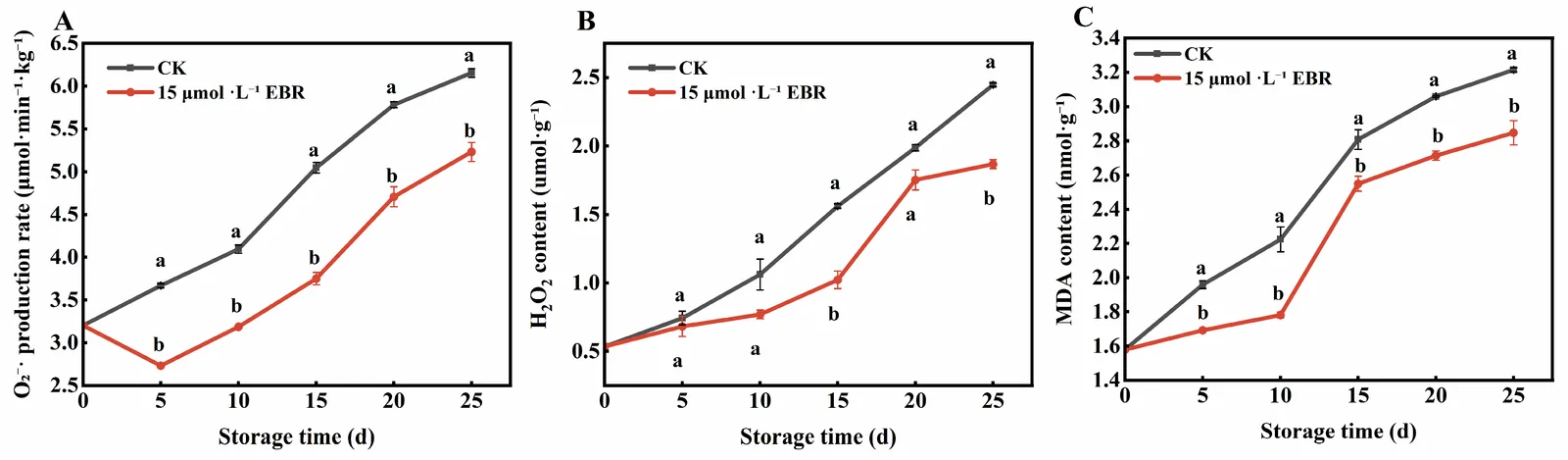
ROS accumulation and oxidative damage during cold storage. (**A**) O_2_•^−^ production rate; (**B**) H_2_O_2_ content; and (**C**) MDA content. Values are expressed as mean ± SE (*n* = 3). Different lowercase letters at the same storage time indicate significant differences between treatments after Bonferroni adjustment (adjusted *p* < 0.05).

**Figure 3:**
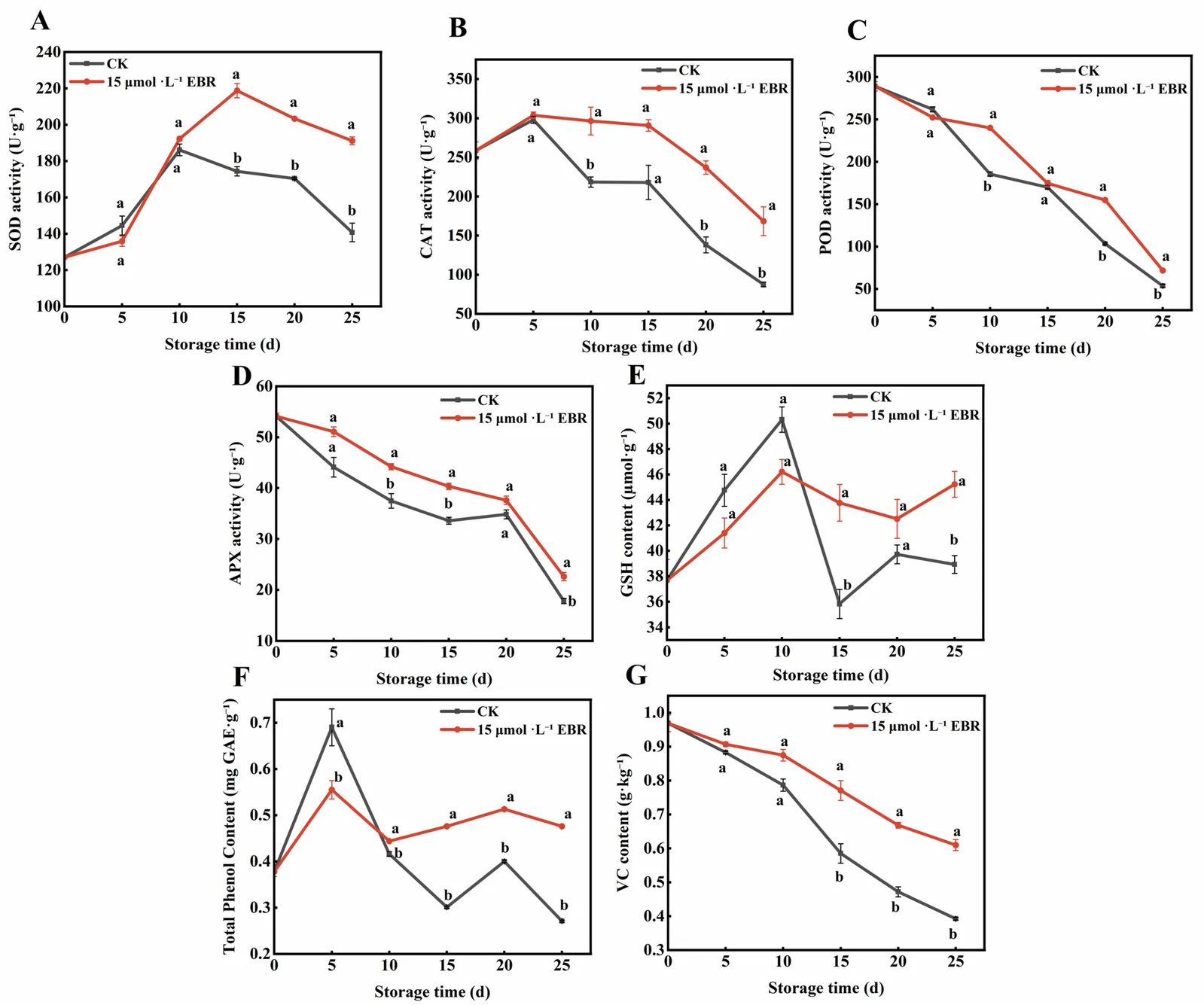
Antioxidant defense responses of green pepper fruit during cold storage. (**A**) SOD activity; (**B**) CAT activity; (**C**) POD activity; (**D**) APX activity; (**E**) GSH content; (**F**) total phenolic content; and (**G**) vitamin C (VC) content. Data are presented as means ± standard errors (*n* = 3). Different lowercase letters at the same storage time indicate significant differences between treatments after Bonferroni adjustment (adjusted *p* < 0.05).

**Figure 4:**
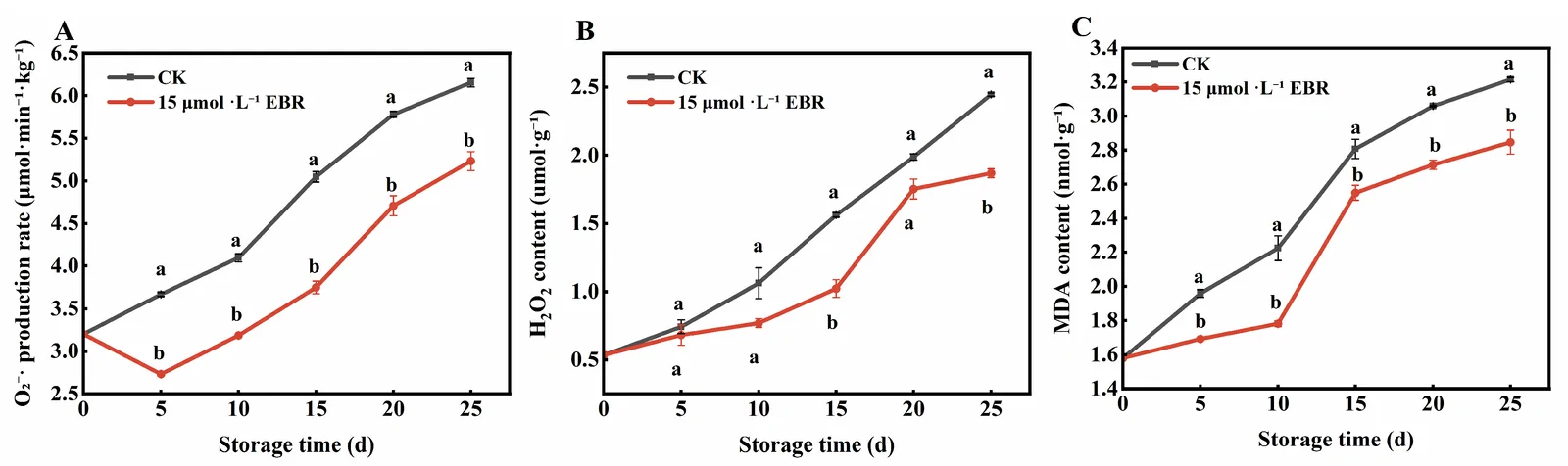
Effects of 24-epibrassinolide on membrane-related parameters in green pepper fruit during cold storage. (**A**) Relative electrical conductivity; (**B**) phospholipase D (PLD) activity; (**C**) lipoxygenase (LOX) activity. Data are presented as the mean ± SE (*n* = 3). Different lowercase letters at the same storage time indicate significant differences between treatments after Bonferroni adjustment (adjusted *p* < 0.05).

**Figure 5:**
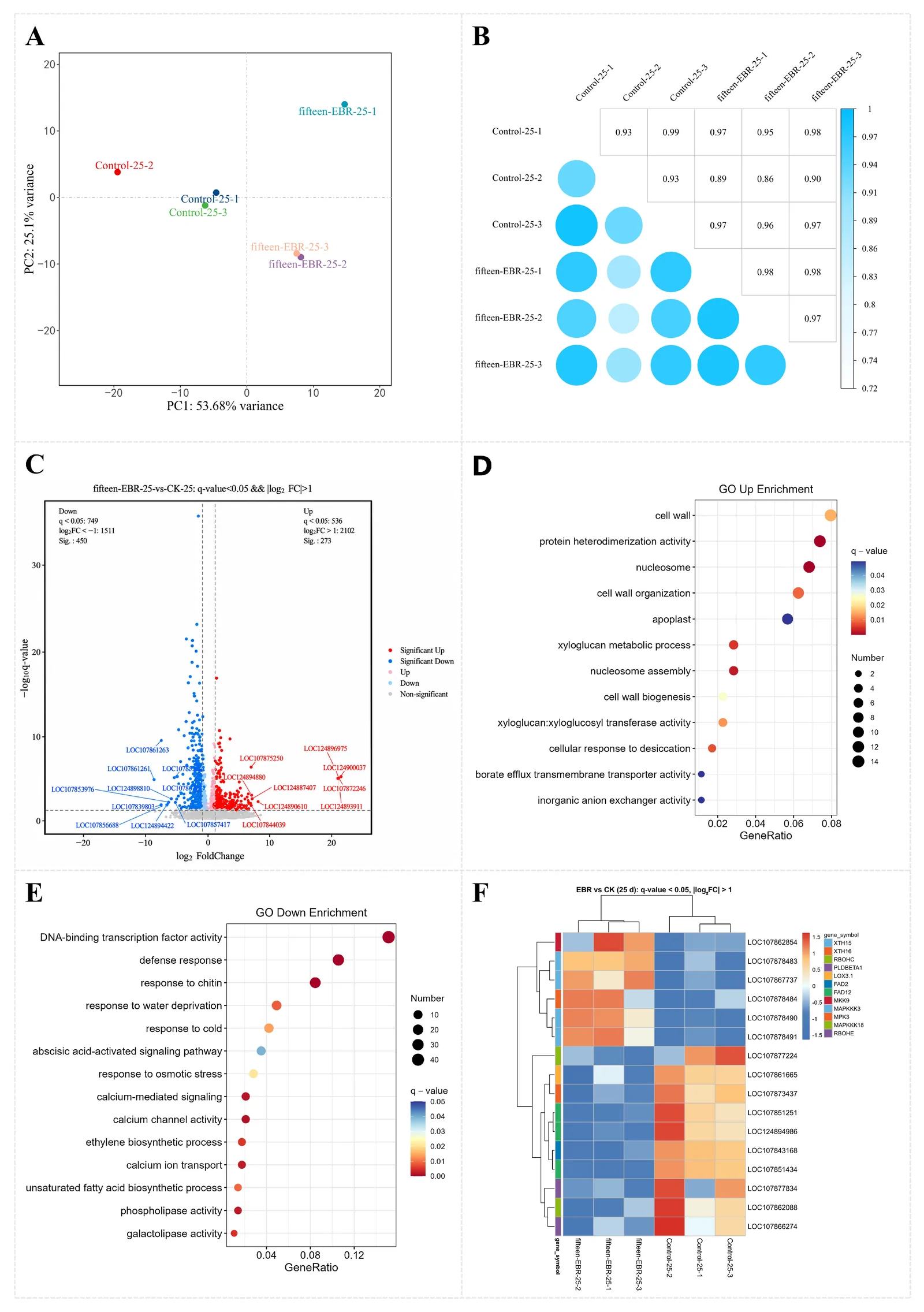
Transcriptomic analysis of green pepper fruit in response to EBR treatment after 25 d of cold storage. (**A**) PCA of transcriptome data. (**B**) Pearson correlation analysis among samples. In panel (**B**), circle size represents the magnitude of the Pearson correlation coefficient, with larger circles indicating stronger correlations. (**C**) Volcano plot of DEGs between CK- and EBR-treated fruit. (**D**) GO enrichment of upregulated DEGs. (**E**) GO enrichment of downregulated DEGs. (**F**) Heatmap of selected candidate genes associated with cell wall remodeling, ROS production, membrane lipid-related processes, and stress signaling. DEGs were defined by padj < 0.05 and |log_2_(fold change)| > 1. Significant GO terms were identified using a BH-adjusted *p*-value < 0.05. The q-value labels retained in panels (**C**–**F**) represent *p*-values adjusted using the Benjamini–Hochberg procedure; for DEG analysis, these values correspond to padj. Note: Control-25-1, Control-25-2, and Control-25-3 represent three biological replicates of the control group stored for 25 d; fifteen-EBR-25-1, fifteen-EBR-25-2, and fifteen-EBR-25-3 represent three biological replicates of the 15 μmol·L^−1^ EBR treatment group stored for 25 d.

## Data Availability

The datasets analyzed during the current study are available from the corresponding author upon reasonable request.

## Supplementary Materials

The following supporting information can be downloaded at: https://www.mdpi.com/article/10.3390/plants15192931/s1, Table S1: Detailed assay conditions and calculation procedures for oxidative stress and antioxidant defense parameters; Table S2: Summary of RNA-seq quality-control metrics of green pepper fruit samples; Table S3: Candidate genes shown in Figure 5F and their differential-expression statistics; Table S4: List of abbreviations used in the manuscript and Supplementary Materials.
