# Supplementary material for: Exogenous 24-Epibrassinolide Alleviates Postharvest Chilling Injury in Green Pepper Fruit Associated with Enhanced Antioxidant Defense and Reduced Membrane Damage
Source: Plants (Basel). 2026 Sep 24;15(19):2931. doi: 10.3390/plants15192931 (PMC13666608; doi:10.3390/plants15192931)
Supplement: Supplementary file 1 [file plants-15-02931-s001.zip › Supplementary Materials.pdf]

Supplementary

Table S1. Detailed assay conditions and calculation procedures for oxidative stress and antioxidant defense parameters.

Parameter	Commercial kit	Sample preparation	Reaction and detection	Calculation / calibration	Final unit
**SOD**	AKAO001M-100S, Boxbio	0.1 g liquid-N_2-ground tissue + 1.0 mL extraction reagent; 8000 × g, 10 min, 4 °C	20 μL extract was used in a 200 μL xanthine/XOD–NBT reaction system; incubation at 25 °C for 30 min; A_{560} measured	$I = (\Delta A_{blank} - \Delta A_{sample})/\Delta A_{blank}$; SOD = $10ID/[(1-I)W]$. One unit corresponds to 50% inhibition of NBT reduction.	$U \cdot g^{-1}$
**POD**	BC0095, Solarbio	0.1 g liquid-N_2-ground tissue + 1.0 mL extraction reagent; 8000 × g, 10 min, 4 °C	Reaction mixture: 120 μL reagent 1, 30 μL reagent 2 working solution, 30 μL reagent 3, 60 μL water, and 5 μL extract; A_{470} recorded at 30 and 90 s	$\Delta A = A_{90s} - A_{30s}$; one unit was defined as an increase of 0.01 in A_{470} min^{-1}. Activity was calculated according to the kit equation on a tissue-mass basis.	$U \cdot g^{-1}$
**CAT**	BC0205, Solarbio	0.1 g liquid-N_2-ground tissue + 1.0 mL extraction reagent; 8000 × g, 10 min, 4 °C	10 μL extract + 190 μL CAT working solution; A_{240} recorded at 5 and 65 s	$\Delta A = A_{5s} - A_{65s}$. Activity was calculated from H_2O_2 decomposition using $\varepsilon_{H2O2} =$ 43.6$L \cdot mol^{-1} \cdot cm^{-1}$ and expressed on a tissue-mass basis; 1 U corresponds to decomposition of 1 μmol H_2O_2 min^{-1}.	$U \cdot g^{-1}$
**APX**	AKVI006M, Boxbio	0.1 g liquid-N_2-ground tissue + 1.0 mL extraction reagent; 12,000 × g, 20 min, 4 °C	20 μL extract + 140 μL reagent 1 + 20 μL reagent 2 + 20 μL reagent 3; A_{290} recorded at 10 and 130 s	After blank correction, $\Delta A = (A_1 - A_2)_{sample} - (A_1 - A_2)_{blank}$; APX = $3.57\Delta A/W$	$U \cdot g^{-1}$
**$O_2 \bullet^-$ production**	BC1295, Solarbio	0.1 g liquid-N_2-ground tissue + 1.0 mL extraction reagent; 12,000 rpm, 10 min, 4 °C	100 μL extract + 80 μL reagent 1, 37 °C for 20 min; then 60 μL reagent 2 + 60 μL reagent 3, 37 °C for 20 min; 100 μL chloroform added, followed by centrifugation at 8000 rpm for 5 min at 25 °C; upper phase measured at 530 nm	A 0.03125 $\mu mol \cdot mL^{-1}$ standard was used. Kit calculation: $O_2 \bullet^-$ production rate = $0.003125\Delta A_{sample}/(\Delta A_{standard}W)$ $\mu mol \cdot min^{-1} \cdot g^{-1}$; values were converted to $\mu mol \cdot min^{-1} \cdot kg^{-1}$.	$\mu mol \cdot min^{-1} \cdot kg^{-1}$
**H_2O_2**	AKAO009M, Boxbio	0.1 g liquid-N_2-ground tissue + 1.0 mL pre-cooled acetone; 8000 × g, 10 min, 4 °C	300 μL extract + 30 μL reagent 2 + 60 μL reagent 3; centrifuged at 8000 × g for 10 min; precipitate dissolved in 300 μL	H_2O_2 standards: 0.25, 0.5, 1.0, 1.5, 2.0, and 2.5 $\mu mol \cdot mL^{-1}$. From $y = kx + b$, x was calculated from sample ΔA; $H_2O_2 = x/W$.	$\mu mol \cdot g^{-1}$

Parameter	Commercial kit	Sample preparation	Reaction and detection	Calculation / calibration	Final unit
			reagent 4 and allowed to stand for 5 min; A_{415} measured		
**GSH**	BC1175, Solarbio	0.1 g liquid-N_2-ground tissue + 1.0 mL extraction reagent; 12,000 × g, 10 min, 4 °C	20 μL extract was reacted with the kit chromogenic reagents for 2 min at room temperature; A_{412} measured by the DTNB method	GSH standards: 25, 50, 100, 200, and 300 $\mu g \cdot mL^{-1}$. Concentration x was obtained from the standard curve; kit result = xF/W $\mu g \cdot g^{-1}$ and was subsequently converted to $\mu mol \cdot g^{-1}$ using the GSH molecular weight (307.32 $g \cdot mol^{-1}$).	$\mu mol \cdot g^{-1}$
**Total phenolics**	AKPL016M-100S, Boxbio	0.1 g tissue + 2.0 mL extraction reagent; sonication at 300 W and 60 °C for 30 min; 12,000 × g, 10 min, room temperature	10 μL extract + 50 μL reagent 1, 2 min at room temperature; then 50 μL reagent 2 + 90 μL water, 10 min; A_{760} measured	Gallic acid standards: 0.0125, 0.025, 0.05, 0.1, 0.2, and 0.3 $mg \cdot mL^{-1}$. From $y = kx + b$, x was obtained; total phenolics = $2x/W$.	mg GAE$\cdot g^{-1}$

Table S2. Summary of RNA-seq quality-control metrics of green pepper fruit samples.

Sample	Raw reads (M)	Clean reads (M)	Clean bases (Gb)	Valid bases (%)	Q30 (%)	GC (%)	Mapping rate (%)	Uniquely mapped reads (%)	RIN
Control-25-1	25.12	24.13	7.03	96.06	94.78	42.73	97.19	94.39	9.1
Control-25-2	25.51	24.06	6.90	94.33	94.52	42.85	97.21	94.50	9.4
Control-25-3	25.04	23.97	6.96	95.76	94.78	42.55	97.19	94.49	9.5
fifteen-EBR-25-1	24.80	23.79	6.92	95.95	94.75	43.05	97.23	94.49	9.5
fifteen-EBR-25-2	25.45	24.29	7.04	95.45	94.62	42.81	97.21	94.54	9.5
fifteen-EBR-25-3	21.87	21.18	6.22	96.88	95.03	42.77	97.21	94.56	9.5

M, million reads; Gb, gigabases; RIN, RNA integrity number.

Table S3. Candidate genes shown in Figure 5F and their differential-expression statistics.

Gene symbol	Capsicum annuum gene ID	Functional annotation	Regulation	log_2(fold change)	Raw p-value	padj
MKK9	LOC107862854	Mitogen-activated protein kinase kinase 9	Up	1.928	9.213E-05	3.934E-03
XTH15	LOC107878483	Xyloglucan endotransglucosylase/hydrolase protein 15	Up	1.693	2.615E-14	2.253E-11
MAPKKK3	LOC107867737	Mitogen-activated protein kinase kinase kinase 3	Up	1.703	2.291E-10	6.658E-08
XTH16	LOC107878484	Probable xyloglucan endotransglucosylase/hydrolase protein 16	Up	2.328	4.273E-04	1.295E-02
XTH15	LOC107878490	Xyloglucan endotransglucosylase/hydrolase protein 15	Up	1.810	4.053E-12	1.686E-09
XTH15	LOC107878491	Xyloglucan endotransglucosylase/hydrolase protein 15	Up	1.587	8.047E-09	1.517E-06
MAPKKK18	LOC107877224	Mitogen-activated protein kinase kinase kinase 18	Down	-1.475	1.542E-04	5.813E-03
LOX3.1	LOC107861665	Linoleate 13S-lipoxygenase 3-1, chloroplastic	Down	-1.223	6.470E-06	4.701E-04
MPK3	LOC107873437	Mitogen-activated protein kinase 3	Down	-1.032	1.087E-07	1.426E-05
FAD12	LOC107851251	Delta(12)-acyl-lipid-desaturase	Down	-2.081	1.554E-10	4.795E-08
FAD12	LOC124894986	Delta(12)-acyl-lipid-desaturase	Down	-2.253	1.161E-10	3.742E-08
FAD2	LOC107843168	Delta(12)-fatty-acid desaturase FAD2	Down	-3.055	3.220E-21	8.631E-18
FAD12	LOC107851434	Delta(12)-acyl-lipid-desaturase	Down	-2.269	9.552E-19	1.570E-15
RBOHE	LOC107877834	Respiratory burst oxidase homolog protein E	Down	-1.966	1.517E-03	3.312E-02
RBOHC	LOC107862088	Respiratory burst oxidase homolog protein C	Down	-1.195	2.899E-05	1.556E-03
PLDBETA1	LOC107866274	Phospholipase D beta 1	Down	-1.138	3.867E-04	1.191E-02

padj, p-value adjusted for multiple testing using the Benjamini–Hochberg procedure.

Table S4. List of abbreviations used in the manuscript and Supplementary Materials.

Abbreviation	Full term
ANOVA	Analysis of variance
APX	Ascorbate peroxidase
BH	Benjamini–Hochberg
CAT	Catalase
CI	Chilling injury
CK	Control
DEGs	Differentially expressed genes
DTNB	5,5′-Dithiobis(2-nitrobenzoic acid)
EBR	24-Epibrassinolide
ELISA	Enzyme-linked immunosorbent assay
FAD	Fatty acid desaturase
GAE	Gallic acid equivalents
GC	Guanine–cytosine content
GO	Gene Ontology
GSH	Glutathione
LOX	Lipoxygenase
MDA	Malondialdehyde
NADPH	Nicotinamide adenine dinucleotide phosphate
NBT	Nitroblue tetrazolium
PCA	Principal component analysis
PLD	Phospholipase D
POD	Peroxidase
Q30	Percentage of bases with a Phred quality score ≥30
RBOH	Respiratory burst oxidase homolog
RIN	RNA integrity number

Abbreviation	Full term
RNA-seq	RNA sequencing
ROS	Reactive oxygen species
RT-qPCR	Reverse transcription quantitative PCR
SE	Standard error
SOD	Superoxide dismutase
TBA	TBA
TSS	Total soluble solids
VC	Vitamin C
XTH	Xyloglucan endotransglucosylase/hydrolase
